# DK-EffiPointMLP: An Efficient 3D Dorsal Point Cloud Network for Individual Identification of Pigs

**DOI:** 10.3390/ani16040590

**Published:** 2026-02-13

**Authors:** Yuhang Li, Nan Yang, Juan Liu, Yongshuai Yang, Shuai Zhang, Jiaxin Feng, Jie Hu, Fuzhong Li

**Affiliations:** 1 College of Software, Shanxi Agricultural University, Jinzhong 030801, China; 2 Department of Basic Sciences, Shanxi Agricultural University, Jinzhong 030801, China

**Keywords:** 3D point cloud, pig-back individual identification, feature fusion, local feature enhancement

## Abstract

Accurate non-contact individual identification of pigs is crucial for their intelligent and efficient management. However, traditional recognition technologies generally suffer from weak local feature expression, feature redundancy, and insufficient channel importance modeling. To address these challenges, this study proposes a novel network model, DK-EffiPointMLP, for individual identification based on 3D dorsal point clouds. The model integrates a Dual-branch Local Feature enhancement module (DLF) and an Efficient Partial Convolution-Residual Refinement module (EffiConv). Specifically, the DLF module adopts a dual-branch structure of KNN and dilated KNN to expand the receptive field, while the EffiConv module combines 1D convolution with the SE mechanism to strengthen key channel modeling. To evaluate the model, a dataset of 10 individual pigs with 8411 samples was constructed. Experimental results show that DK-EffiPointMLP achieves accuracies of 96.86% on this self-built dataset and 95.2% on ModelNet40. When re-training all baseline models under the same pipeline and preprocessing protocols, our model outperformed existing mainstream models by 2.74 and 1.1 percentage points, respectively. This approach provides an efficient solution for automated management in commercial farming.

## Simple Summary

This study addresses the challenge of non-contact individual pig identification in precision livestock farming. We developed a 3D point cloud recognition model based on PointMLP, enhancing its feature extraction and information processing capabilities. Our model outperforms existing methods, achieving 96.86% accuracy on a dataset of 8411 samples from 10 pigs. This solution enables automated, individual-level management in commercial farms, improving operational efficiency and supporting animal welfare.

## 1. Introduction

Precise individual identification serves as the foundational data pillar for achieving refined management and automated production in modern livestock farming [1,2,3,4]. Given the limitations of existing physical tags and RFID technology in terms of stability and animal welfare [5,6,7], the development of stable and reliable non-contact identification methods is imperative [8]. Within swine farming contexts, individual identification techniques based on dorsal characteristics have garnered significant attention due to their unique advantages [9]. In practical deployment, our system utilizes overhead cameras mounted directly above feeding alleys within a semi-enclosed facility. This setup specifically leverages the natural queuing of pigs during feeding to obtain unobstructed dorsal visibility, effectively mitigating mutual occlusions common in group-housing. To ensure operational robustness, experimental data were captured under fluctuating natural lighting—both with and without direct sunlight—strictly avoiding any external artificial light sources to simulate authentic commercial environments. By precisely capturing stable features such as spinal curvature and body surface undulations, this approach not only overcomes the shortcomings of traditional methods but also provides an efficient, low-interference solution for intelligent management in large-scale pig farms.

With the advancement of three-dimensional perception devices and deep learning techniques [10,11,12,13,14], point cloud-based individual recognition of livestock and poultry has garnered increasing attention [15,16,17,18,19,20]. The PointNet and PointNet++ models proposed by Qi et al. [21,22] provide an end-to-end learning framework for point cloud analysis. PointNet++ effectively enhances the modelling capability of local geometric structures through hierarchical sampling and local feature aggregation mechanisms, laying a methodological foundation for subsequent point cloud applications. In the field of livestock monitoring, research using point clouds generally follows two distinct technical routes: (i) the segmentation of dorsal regions to isolate animals from complex backgrounds, and (ii) the extraction of identity-specific geometric features for individual identification. While the former focuses on spatial localization, the latter—which is the focus of this study—demands higher sensitivity to subtle surface variations. For instance, Zhou et al. [23] employed PointNet++ for the segmentation and feature extraction of pig dorsal point clouds. By refining the local geometric grouping strategy, they developed the PointNet++–LGG model, achieving automated pig identification with high accuracy. This study validated the feasibility of using dorsal geometry for recognition; however, its approach remains constrained by single-scale neighbourhood construction, limiting adaptability to complex pose variations and uneven point cloud density—factors that can significantly degrade identification precision in commercial settings. Billah et al. [24] similarly employed the PointNet++ framework to detect and segment the dorsal region from three-dimensional point clouds in dairy cattle. This approach was applied to tasks including individual identification, lameness detection, and body condition scoring, demonstrating the versatility of point cloud-based dorsal features across multiple livestock perception tasks. However, the model architecture remains relatively complex, exhibiting sensitivity to computational resources and parameter tuning.

Beyond individual recognition, certain studies have extended point cloud deep learning methods to tasks such as object detection and pose estimation. Chen et al. [25] proposed a point cloud pose estimation method integrating PointNet++ with Point Pair Features (PPF), employing deep learning for object detection and combining adaptive density clustering for point cloud segmentation. Subsequently, an enhanced PPF algorithm was utilised to achieve high-precision 6D pose estimation. This approach demonstrates robust geometric modelling capabilities in complex scenes, though its overall workflow remains intricate, posing challenges for direct deployment in real-time-demanding livestock production settings. In recent years, researchers have explored MLP-based modelling methods to enhance efficiency. For instance, Ma et al. [26] proposed PointMLP, simplifying the complex local structures [27] of traditional networks into stacked residual multilayer perceptrons. While PointMLP achieves competitive performance on standard benchmarks, its effectiveness in scenarios requiring fine-grained morphological discrimination—such as identifying visually similar pigs—remains systematically unexplored. Based on these observations, this study is grounded in the hypothesis that integrating multi-scale neighbourhood aggregation with channel-efficient attention can significantly enhance the model’s ability to capture subtle surface undulations on pig backs, thereby improving discrimination among highly similar individuals. Consequently, we aim to address the following evaluation questions: (i) whether a multi-scale MLP architecture can maintain high identification accuracy despite uneven point cloud densities, and (ii) to what extent the optimized channel efficiency reduces the computational overhead for edge deployment in commercial farms.

In summary, existing PointNet++ based approaches have achieved some progress in pig back individual recognition tasks, yet they generally suffer from issues such as fixed local neighbourhood scales, feature redundancy, and inadequate modelling of channel importance. While emerging MLP-type point cloud networks offer advantages in efficiency and structural simplicity, their structural adaptability and performance potential for livestock individual recognition tasks warrant further investigation. To address these issues, this paper proposes DK-EffiPointMLP, a point cloud-based individual recognition method for pigs. This approach aims to overcome the limitations of existing methods, enhance the accuracy and efficiency of pig individual recognition, and provide technical support for non-contact precision identification and intelligent management. The contributions of our method are as follows:

- Contribution 1: To address the limitations of existing livestock individual recognition methods—specifically fixed local neighborhood scales, insufficient feature representation, and poor structural adaptability—we propose DK-EffiPointMLP, a specialized network for individual pig identification based on 3D back point clouds. Leveraging the PointMLP backbone, our approach incorporates two core innovative modules: the Dual-Layer Feature (DLF) module for feature enhancement and the Efficient Convolution and Residual Refinement (EffiConv) module for high-efficiency processing.
- Contribution 2: To address the limitations of fixed local neighborhood scales and limited geometric representation in existing methods, we introduce the Dual-Layer Feature (DLF) module. By employing a dual-branch strategy that runs K-Nearest Neighbors (KNN) and Dilated KNN in parallel, this module fuses multi-scale geometric information. This design effectively expands the receptive field and enriches local feature diversity while preserving local geometric consistency.
- Contribution 3: To tackle the issues of feature redundancy and inadequate channel importance modeling, we design the Efficient Convolution and Residual Refinement (EffiConv) module. This module integrates Partial 1D Convolution (PConv1D) with the Squeeze-and-Excitation (SE) channel attention mechanism [28]. It prioritizes key channel feature extraction while suppressing redundant information, thereby enhancing feature representational capability while simultaneously reducing computational overhead.

## 2. Materials and Methods

Within the domain of intelligent agricultural sensing, balancing recognition accuracy with inference efficiency under constrained computational resources remains a central challenge in model design. Saleem et al. [29] highlighted that optimising network architecture is a key approach to balancing performance and computational cost. Similarly, prior studies have demonstrated that channel redundancy suppression strategies in three-dimensional data can effectively enhance model compactness [30,31]. However, the direct application of generalised backbone networks to individual pig identification remains significantly constrained. Specifically, standard networks often retain substantial redundant channels during deep feature propagation; this not only wastes computational resources but also increases susceptibility to environmental noise interference. Furthermore, existing architectures tend to favour mechanisms for global shape description, frequently overlooking critical local geometric details such as ridge curvature and shoulder undulations. This results in insufficient fine-grained discrimination capabilities when processing visually highly similar pig individuals. To address these issues, we propose DK-EffiPointMLP. This framework integrates optimised feature extraction and convolution modules into the PointMLP architecture. While preserving the overall network structure, it refines the local feature extraction process specifically to meet the practical demands of pig back point cloud individual recognition, ensuring both efficiency and accuracy.

### 2.1. Network Architecture

**Figure 1:**
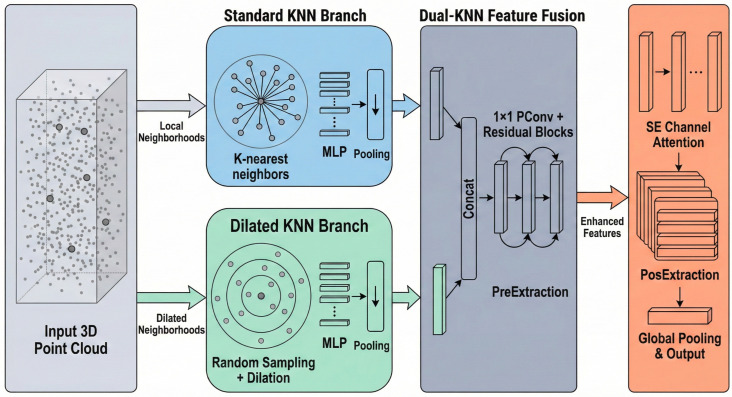
The overall network architecture of DK-EffiPointMLP.

The overall network architecture of DK-EffiPointMLP is illustrated in Figure 1 and detailed in Table 1. For the input raw 3D point cloud *X* (whose shape is denoted as $X\in R^{N\times 3}$, where *N* represents the number of points in the point cloud), each processing stage of the network proceeds as follows:

First, the parallel k-NN branch and the Stochastic Subsampled k-NN branch generate local neighbourhoods with differing receptive fields through distinct construction strategies. The former utilizes standard k-NN to establish a fundamental local neighbourhood, while the latter introduces a random shift sampling mechanism to expand the receptive field. Specifically, this random shift is applied in the coordinate space. Prior to the neighbor search, a random translation offset is added to the coordinates of the query points, which is sampled from a uniform distribution within the range [−δ,δ] (typically set to δ=0.2). This mechanism enables the model to capture richer local geometric information by introducing stochasticity into the neighborhood construction.

To mitigate redundancy, Partial Convolutions (PConv) and channel attention mechanisms are incorporated within the residual blocks of the module, thereby optimizing feature extraction efficiency. Specifically, for the PConv1D layer, we set the partial ratio to 1/4 (r=1/4). This means that only the first 1/4 of the input channels participate in the spatial convolution to extract spatial features, while the remaining 3/4 channels pass through unchanged to retain the original information. This ratio is kept constant across all layers to achieve a balance between reducing floating-point operations (FLOPs) and maintaining feature diversity.

Finally, the local features are fed into the SE attention mechanism to enhance feature extraction. This module employs a global average pooling layer to aggregate global spatial information, followed by two fully connected (FC) layers to model channel dependencies. Specifically, the reduction ratio *r* is set to 8, which reduces the channel dimension in the first FC layer to C/8 to limit model complexity. A ReLU activation function is applied between the two FC layers to introduce non-linearity, while a Sigmoid function is used after the second FC layer to generate the final channel weights. This step globally recalibrates the features, yielding refined representations that provide robust support for subsequent tasks.

### 2.2. Dual-Branch Feature Enhancement Module (DLF Module)

**Figure 2:**
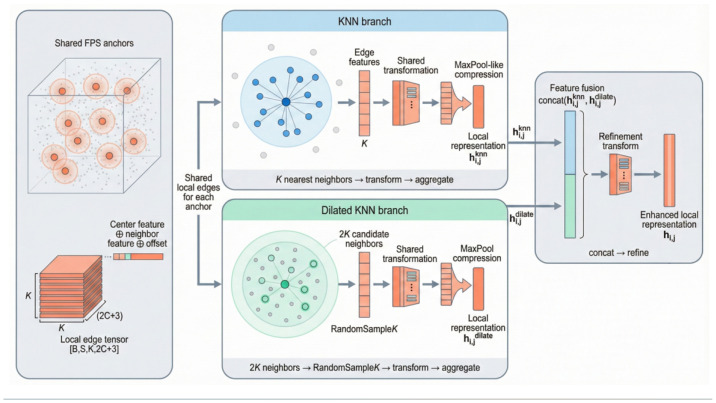
Diagram of the geometric neighbourhood construction.

To enhance the expressive power of local features in point clouds while maintaining computational efficiency, this paper designs a dual-branch feature enhancement module at the local neighbourhood level. This module comprises a KNN branch and an extended KNN branch: the KNN branch selects the K nearest neighbours for each centre point, while the extended KNN branch first retrieves 2K nearest neighbours and then randomly selects K points from them to introduce a more diverse distribution of neighbourhood samples. By synergistically fusing the local representations from both branches within the feature space, this module maintains consistency and stability in local geometric relationships while effectively enhancing the diversity and information density of neighbourhood features. This mitigates issues such as feature sparsity and limited expressive power commonly encountered with traditional single KNN in complex point cloud scenarios, thereby comprehensively strengthening the network’s ability to model local geometric structures and its robustness against noise perturbations. The overall structure of this module is illustrated in Figure 2.

Specifically, the local neighborhood features extracted from the standard k-NN branch and the Stochastic Subsampled k-NN branch are concatenated along the neighbor dimension. Since each branch contributes K neighbors, the fused neighborhood contains a total of 2K points for each center point. Consequently, the input data is transformed into a unified local feature representation with the shape [B, S, 2K, C]. This strategy enables the model to simultaneously leverage the dense local information from the standard k-NN and the expanded receptive field from the stochastic branch. Unless otherwise specified, the value of K is set to 20 in our experiments. During the forward propagation, this aggregation process is formalized as shown in Equation (1):

(1) $$h_{i,j}=\mathrm{concat}\left(h_{i,j}^{\mathrm{knn}},h_{i,j}^{\mathrm{dilate}}\right),\;j=1,\ldots ,2K,$$

where $h_{i,j}^{\mathrm{knn}}$ and $h_{i,j}^{\mathrm{dilate}}$ denote the local neighbourhood features derived from the KNN branch and the Dilated KNN branch, respectively.

#### 2.2.1. KNN Branch

In the KNN branch, to enhance the representational capability of local geometric features within the point cloud, we systematically introduce the KNN algorithm. Consider a point cloud set $X=\{x_{j}|j=1,\ldots ,N\}$ with $X\in R^{N\times C}$, where each point is represented by a C-dimensional feature vector encompassing 3D coordinates (x,y,z) and other attribute features. Focusing on any arbitrary point $x_{i}$ as the center, we calculate the Euclidean distance between it and all other points in the set, as defined in Equation (2):

(2) $$D=d\left(x_{i},x_{j}\right)={∥x_{i}-x_{j}∥}_{2},$$

Subsequently, we calculate the distances to all other points in the point cloud and select the nearest K points to constitute the local neighborhood set $N_{i}$, as formulated in Equation (3):

(3) $$N_{i}=\mathrm{topK}\left(D\right),$$

where *X* represents the point cloud data; *N* and *C* denote the total number of points and the feature dimension of each point, respectively; topK(·) indicates the operation of selecting the K nearest neighbors; and d(·,·) serves as the distance function (specifically, Euclidean distance in this study).

From a geometric perspective (as illustrated in the upper part of Figure 2), this process is equivalent to constructing a local spherical neighbourhood centered at $x_{i}$ in the 3D space. Within this “sphere”, the nearest K sample points are retained for subsequent local feature aggregation and representation learning.

Upon completion of the neighborhood construction, the features $X_{N_{i}}$ of all neighboring points are aggregated into a local representation, denoted as $h_{i,j}^{\mathrm{knn}}$, as calculated in Equation (4):

(4) $$h_{i,j}^{\mathrm{knn}}=\mathrm{aggregate}(X_{N_{i}}),$$

where aggregate(·) denotes the feature aggregation operation. Specifically, regarding implementation, the center point features, neighbor features, and their relative positions are concatenated along the channel dimension to form edge features. Following a nonlinear transformation via a shared MLP, channel-wise max-pooling is applied across the neighbor dimension to yield a permutation-invariant aggregated representation of the local neighborhood.

While this method effectively captures the local geometric structure of point clouds, it encounters limitations in complex scenes. Specifically, KNN may result in sparse and inadequate feature representation, particularly when point clouds contain noise or exhibit intricate structures, as the neighbourhood selection mechanism of KNN proves overly restrictive.

#### 2.2.2. Expand KNN Branches

In the Dilated KNN branch, we introduce an “expansion + random sampling” mechanism based on the KNN branch to further enrich the feature diversity of the local neighbourhood. We continue to denote a point cloud frame as formulated in Equation (5):

(5) $$X=\left\{x_{j}|j=1,\ldots ,N\right\},\;X\in R^{N\times C},$$

where *X* represents the entire point cloud data, *N* denotes the total number of points, and *C* signifies the feature dimension of each point. For any arbitrary center point $x_{i}$, the Euclidean distance is calculated similarly, as defined in Equation (6):

(6) $$\tilde{D}=d\left(x_{i},x_{j}\right)={∥x_{i}-x_{j}∥}_{2},$$

Subsequently, we calculate the distances to all other points in the point cloud. Unlike the standard KNN branch, which directly selects the nearest K neighbours, the Dilated KNN branch first selects the nearest 2K candidate neighbours, denoted as ${\tilde{N}}_{i}$, as formulated in Equation (7):

(7) $${\tilde{N}}_{i}=\mathrm{top}(2K)\left(\tilde{D}\right),$$

where top(2K)(·) denotes the operation of selecting the indices of the nearest 2K points based on distance in ascending order. Following this, random subsampling is performed on the candidate set ${\tilde{N}}_{i}$ to obtain the final dilated neighbourhood $N_{i}^{\mathrm{dilate}}$, as shown in Equation (8):

(8) $$N_{i}^{\mathrm{dilate}}={\mathrm{RandomSample}}_{K}({\tilde{N}}_{i}),$$

where ${\mathrm{RandomSample}}_{K}\left(\cdot \right)$ represents the random sampling of *K* elements from a given set.

From a geometric perspective (as illustrated in the lower part of Figure 2), this process is equivalent to constructing a local spherical neighbourhood centred at $x_{i}$, initially forming a 2K neighbourhood shell, and then randomly selecting K neighbour points from within it. This mechanism makes the neighbourhood distribution more diverse.

Upon completion of the neighbourhood construction, the features $X_{N_{i}^{\mathrm{dilate}}}$ of all neighbour points within the neighbourhood are aggregated into a local representation, denoted as $h_{i,j}^{\mathrm{dilate}}$, as defined in Equation (9):

(9) $$h_{i,j}^{\mathrm{dilate}}=\mathrm{aggregate}(X_{N_{i}^{\mathrm{dilate}}}).$$

By introducing the “2K candidates + random sampling” strategy, the Dilated KNN branch significantly enhances the diversity of neighbourhood features while preserving the perception of local geometric structures. This contributes to obtaining more robust local representations in point cloud scenes characterized by noise and complex structures.

### 2.3. Efficient Partial Convolution and Residual Refinement Module (EffiConv Module)

**Figure 3:**
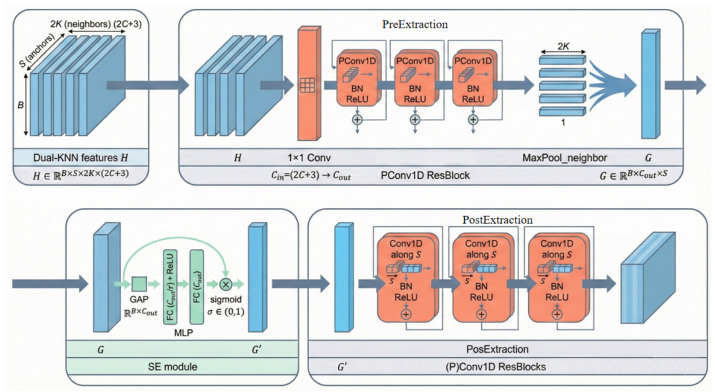
The overall architecture of the EffiConv module.

Although the Dual-Branch Feature Enhancement module significantly improves the model’s feature extraction capability, potential remains for further optimisation, particularly in capturing fine-grained features within complex pig farming scenarios. To address this challenge, this paper introduces the EffiConv module. This module integrates Partial 1D Convolutions (PConv1D) [32] with residual refinement structures (PreExtraction and PosExtraction) to maintain robust feature extraction capabilities. Specifically, PConv1D optimises efficiency by performing convolutions solely on selected input channels while leaving the remaining channels unchanged. Complementarily, the residual refinement mechanism synergistically captures multi-scale contextual information and local details through a pre-extraction and post-integration strategy. This design mitigates potential information loss induced by partial convolutions, thereby achieving an effective balance between computational efficiency and performance. The overall architecture of the EffiConv module is illustrated in Figure 3.

#### 2.3.1. PConv1D ResBlock

**Figure 4:**
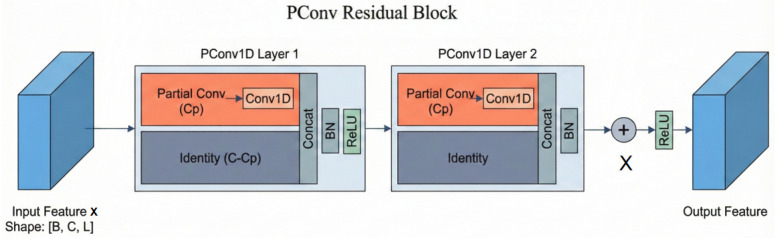
Illustration of the convolutional residual blocks in PointMLP.

In the PointMLP architecture, local feature extraction primarily relies on multi-layer convolutional residual blocks, as illustrated in Figure 4.

The proposed PConv1D ResBlock comprises two cascaded PConv1D layers connected by a residual skip connection. Within each PConv1D layer, to achieve efficient feature extraction, the input feature channels are divided into two mutually exclusive subsets: one constituting the computational activation region (indicated by the orange area in Figure 4) which participates in the convolution operation, and the other serving as the feature retention region that passes input features directly to the next layer. This mechanism effectively prevents information decay caused by feature transformation. Specifically, the network performs one-dimensional convolution solely on the activation region channels to capture local geometric patterns, whilst the preservation region channels bypass computation to reuse historical feature information. Subsequently, the concatenated features undergo Batch Normalisation (BN) to unify their scale, followed by ReLU activation. Finally, the deep features processed through two PConv1D layers are fused with the original input via element-wise addition. This approach significantly reduces computational redundancy while effectively preserving the integrity of critical local features (such as curvature and edges) in point cloud geometric feature extraction tasks.

Based on the aforementioned principles, PConv significantly reduces computational complexity while effectively capturing spatial structural information, rendering it particularly well-suited for processing data exhibiting strong local correlations. This characteristic confers a distinct advantage in point cloud processing tasks for pig dorsal regions, where spatial features within local neighbourhoods often exhibit high redundancy. Consequently, PConv excels at extracting local geometric patterns at minimal computational cost. Within the DK-EffiPointMLP network proposed in this paper, PConv replaces the standard convolution operation within the residual blocks. Specifically, when the number of input and output channels remains consistent, only a subset of channels undergoes convolution modelling, whilst the remaining channels are directly retained for subsequent feature fusion. Conversely, when the channel count changes, the architecture adopts a conventional convolution structure to ensure network stability and generalisability. This strategy significantly reduces computational redundancy while preserving feature expressiveness without altering the original network topology, making it highly effective for local feature extraction in point clouds.

#### 2.3.2. SE Channel Attention Bolck

**Figure 5:**
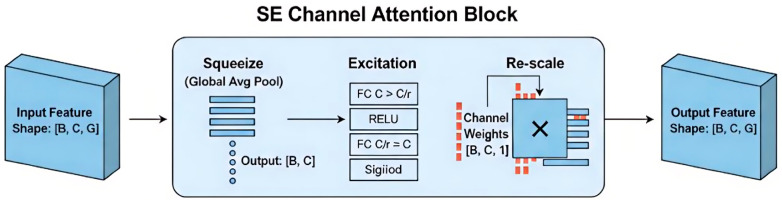
Structure of the SE channel attention module.

Following the introduction of the KNN branch and the dual-neighbourhood KNN branch, significant variations are observed in the importance of different channels for characterising individual geometric morphology and local structural information. To further enhance feature discriminability, an SE channel attention mechanism is introduced following local feature extraction at each stage, enabling the adaptive recalibration of channel features. The structure of the SE module is illustrated in Figure 5.

To enhance the model’s sensitivity to critical feature channels, the constructed SE channel attention module primarily comprises three core stages: Squeeze, Excitation, and Re-scale. Initially, the Squeeze stage employs Global Average Pooling to aggregate input features across the spatial dimension, generating channel descriptors containing global contextual information. Subsequently, the Excitation stage utilises a two-layer fully connected network—incorporating dimensionality reduction (C→C/r) and expansion (C/r→C)—to capture non-linear dependencies between channels. A Sigmoid function is then applied to generate normalised channel weights. Finally, in the Re-scale stage, channel-wise scaling is performed on the original input features of the SE module. This process involves broadcasting the weight vector and multiplying it element-wise with the input feature map, thereby reinforcing effective feature representations while suppressing the influence of low-response channels.

In the network architecture presented in this paper (as illustrated in Figure 1), the SE module is integrated into the feature extraction process at each stage. Positioned subsequent to local feature aggregation and prior to positional feature extraction, it performs channel-level filtering and enhancement on features processed by the Dual-Branch Feature Enhancement module. This design effectively enhances the model’s perception of individual geometric variations, thereby improving classification stability and robustness in point cloud individual recognition tasks.

Collectively, the PConv Residual Block and the SE Channel Attention Block constitute complementary feature enhancement mechanisms within the network. From the perspective of computational efficiency, PConv mitigates redundant channel convolutions, thereby streamlining the feature extraction process. Conversely, from the perspective of feature representation, the SE module adaptively allocates finite channel resources, enhancing the utilisation of salient features. Their synergistic interaction enables the DK-EffiPointMLP network to achieve stable local feature extraction without compromising model expressiveness, yielding a robust feature foundation for subsequent point cloud individual recognition.

### 2.4. Implementation Details

The proposed DK-EffiPointMLP was implemented using the PyTorch framework on an NVIDIA RTX 4090 D GPU. To ensure the reproducibility of our experiments, the random seed was fixed at 42. The input point clouds were normalized to the unit sphere (centered at the origin and scaled).

During the training phase, we employed the Stochastic Gradient Descent (SGD) optimizer with a momentum of 0.9 and Nesterov acceleration set to True. The initial learning rate was set to 0.0015 and dynamically adjusted using a Cosine Annealing scheduler with a minimum learning rate of $1\times 10^{-5}$. We utilized the Cross-Entropy loss function combined with a label smoothing factor of 0.1 to mitigate overfitting.

Data augmentation techniques were applied to enhance the model’s robustness, including random scaling with a ratio in the range of [0.8, 1.2], random translation within [−0.1, 0.1], and point shuffling. The model was trained for 150 epochs with a batch size of 16. An early stopping mechanism was implemented to terminate training if the validation accuracy did not improve for 20 consecutive epochs.

## 3. Results

To evaluate the performance of DK-EffiPointMLP for individual pig recognition based on dorsal point clouds, this section first outlines the experimental environment, dataset construction, and evaluation metrics. Subsequently, we benchmark the proposed model against mainstream methods, analyze the effectiveness of its core modules, quantify the contribution of each component via ablation studies, and investigate the sensitivity of key parameters.

### 3.1. Experimental Environment

To ensure reproducibility and fair comparison, all models were trained and tested within an identical hardware and software environment. The specific configurations and hyperparameters are detailed in Table 2.

### 3.2. Experimental Data

The study dataset was collected from Huifeng Livestock Breeding Professional Cooperative (Guangling, Shanxi) and other small-and-medium-sized commercial pig farms, with the researcher obtaining oral informed consent from all pig farm owners (legal animal owners) prior to data collection. Farmers were fully informed of the research objectives, procedures and data use scope, and voluntarily agreed to participate without concealment, misleading or coercion. This study adopted a non-intrusive design, and the shooting of camera equipment will not interfere with the daily activities of pigs, avoiding impacts on routine feeding management and ensuring data ecological validity. It followed the no-contact principle with purely non-invasive observation, no direct intervention, physical contact or environmental changes to pigs, complying with domestic and international animal research ethics and thus exempt from IRB approval. The study’s ethical compliance was reviewed and confirmed by the Institutional Review Board of the College of Software, Shanxi Agricultural University.

#### 3.2.1. Data Collection

**Figure 6:**
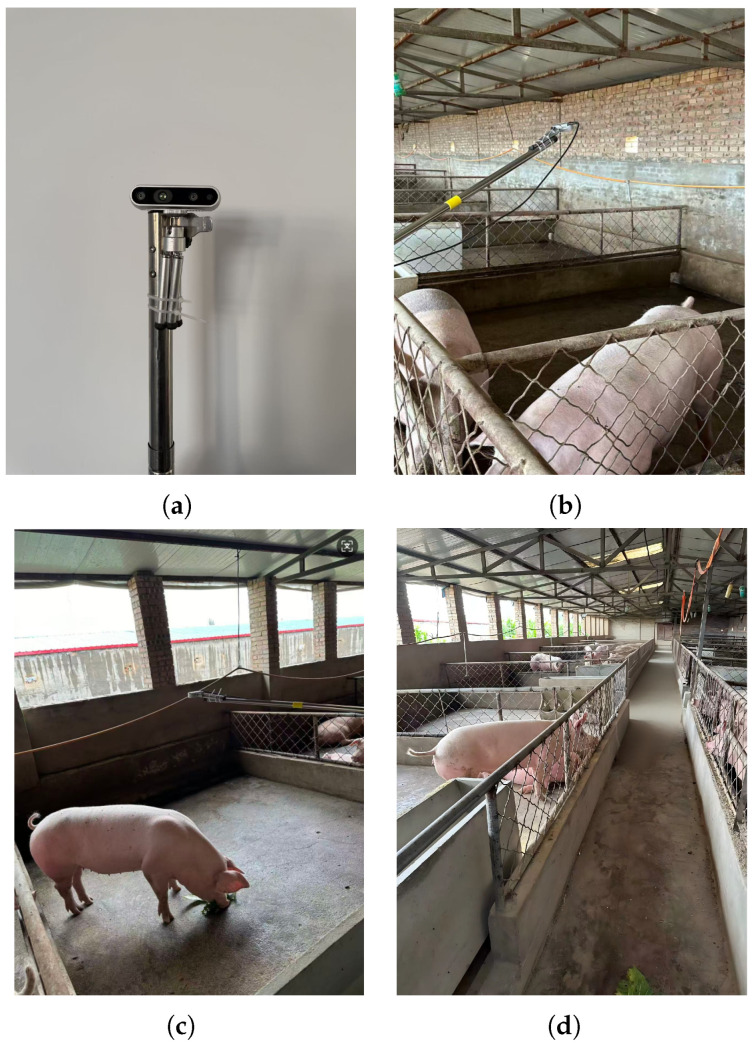
Data acquisition process. (**a**) Intel RealSense D435i depth camera. (**b**) Top-down mounting configuration. (**c**) Data collection under natural illumination. (**d**) Data collection in shaded conditions.

The experimental subjects consisted of ten healthy commercial pigs of similar age, body weight, and uniform coat color, selected to minimize interference from non-geometric characteristics. Data were collected using an Intel RealSense D435i stereo depth camera mounted on an extendable pole in a top-down configuration, as illustrated in Figure 6a. Depth information was generated based on stereo vision principles [16,17,33]. Data acquisition was conducted under two scenarios: natural illumination and shaded conditions without direct sunlight (excluding artificial supplementary lighting). In total, ten BAG-format video segments were obtained with a resolution of 468×828 pixels and a frame rate of 30 Hz. Each video segment lasted approximately 90 s, capturing continuous sequences of individual pigs. After filtering for quality—removing frames with severe motion blur or incomplete dorsal coverage—a total of 8411 single-frame point clouds were retained. This yielded a balanced dataset with approximately 840 samples per pig across the ten categories. To ensure data independence, these samples were partitioned into training and testing sets at an 8:2 ratio using a video-level split, ensuring that no frames from the same 90-s recording session appeared in both subsets (Figure 6).

#### 3.2.2. Data Preprocessing

**Figure 7:**
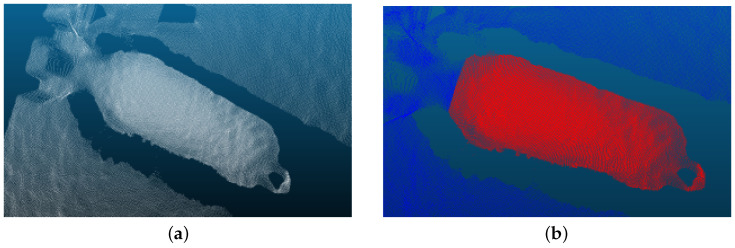
Point cloud preprocessing. (**a**) The generated raw 3D point cloud of the pig body. (**b**) The retained dorsal region point cloud after removing the background (ground, fences) and head using CloudCompare.

First, the BAG files were parsed using the Intel RealSense SDK to synchronously extract aligned depth and RGB frames. Manual filtering was subsequently applied to exclude frames containing occlusions, motion blur, or depth measurement anomalies, retaining only valid frames with distinct dorsal contours. By incorporating the camera intrinsic parameters (focal lengths $f_{x},f_{y}$ and principal point coordinates $C_{x},C_{y}$), the depth value *Z* of each pixel (u,v) was projected into the camera coordinate system to generate the 3D point cloud (X,Y,Z), as shown in Figure 7.

Subsequently, the point cloud processing tool CloudCompare (version 2.13.0) was employed to manually annotate background elements, including the ground, fences, and pig head. To ensure the reliability of this process, we identified the curvature mutation points based on the sharp transition in surface geometry between the neck and the shoulder blades. This manual trimming followed a standardized protocol to ensure consistency; a reliability check showed that samples processed by different annotators maintained high geometric consistency. By using these objective anatomical landmarks, the dorsal region point cloud was separated and retained, minimizing subjective bias and ensuring the extracted features are representative of the individual’s morphology, as illustrated in Figure 7b.

Each single-frame dorsal point cloud represented one sample and was stored as a .txt file containing three-dimensional coordinates. Utilizing a customized PigDataset class compliant with the PyTorch Dataset interface, the data were uniformly resampled to 2048 points, employing random sampling with replacement when the point count was insufficient. This process yielded a total of 8411 samples. To strictly prevent temporal leakage and ensure data independence, the dataset was partitioned into a training set (6725 samples) and a test set (1686 samples) at a ratio of 8:2 based on independent video sequences. By ensuring that all frames from the same continuous recording session were assigned exclusively to either the training or the testing set, we eliminated the risk of accuracy inflation caused by near-duplicate adjacent frames. A balanced distribution of the ten individual pig categories was maintained across both subsets to ensure the fairness of the evaluation.

Furthermore, during the training phase, data augmentation techniques [34] were applied, including random scaling (factor: 0.8–1.2), translation (displacement: ±0.1), and random point reordering. Rotation augmentation was excluded because the dorsal point clouds are gravity-aligned by the fixed overhead cameras, maintaining a consistent vertical orientation. To ensure a fair comparison, these augmentation strategies were applied identically to all baseline models.

### 3.3. Evaluation Indicators

Overall Classification Accuracy (OA) and Mean Class Accuracy (mAcc), which are standard metrics in point cloud classification tasks, were selected as the core evaluation indicators. These metrics allow for the assessment of model performance across two dimensions: overall efficacy and class balance.

The Overall Classification Accuracy (OA) measures the model’s comprehensive recognition capability across all test samples. The calculation is performed as shown in Equation (10):

(10) $$OA=\frac{N_{\mathrm{correct}}}{N_{\mathrm{total}}},$$

where $N_{\mathrm{correct}}$ denotes the total number of correctly predicted samples, and $N_{\mathrm{total}}$ represents the total number of samples in the test set.

The Mean Class Accuracy (mAcc) is employed to mitigate the impact of class imbalance and reflects the average recognition performance for each individual pig. It is defined in Equation (11):

(11) $$mAcc=\frac{1}{C}\sum_{i=1}^{C}\frac{N_{\mathrm{correct},i}}{N_{\mathrm{total},i}},$$

where C=10 represents the number of individual pig categories, while $N_{\mathrm{correct},i}$ and $N_{\mathrm{total},i}$ denote the number of correctly predicted samples and the total number of samples for the *i*-th class, respectively.

### 3.4. Performance Comparison

To validate the performance advantages of DK-EffiPointMLP, representative models in the point cloud classification domain, including PointNet, PointNet++, and PointMLP, were selected as comparative benchmarks. All models were trained and tested under identical experimental settings, and the results are presented in hyperparameters are detailed in Table 3.

Under identical datasets and consistent training configurations, the proposed DK-EffiPointMLP achieved optimal performance in pig individual recognition tasks, attaining an overall accuracy (OA) of 96.86%. Compared to the classical point cloud methods PointNet and PointNet++, the OA improved by 7.94 and 3.93 percentage points respectively. Relative to the baseline backbone network PointMLP, the accuracy also increased by 2.74 percentage points. Concurrently, DK-EffiPointMLP demonstrated consistent superiority in the mean accuracy (mAcc) metric.

**Figure 8:**
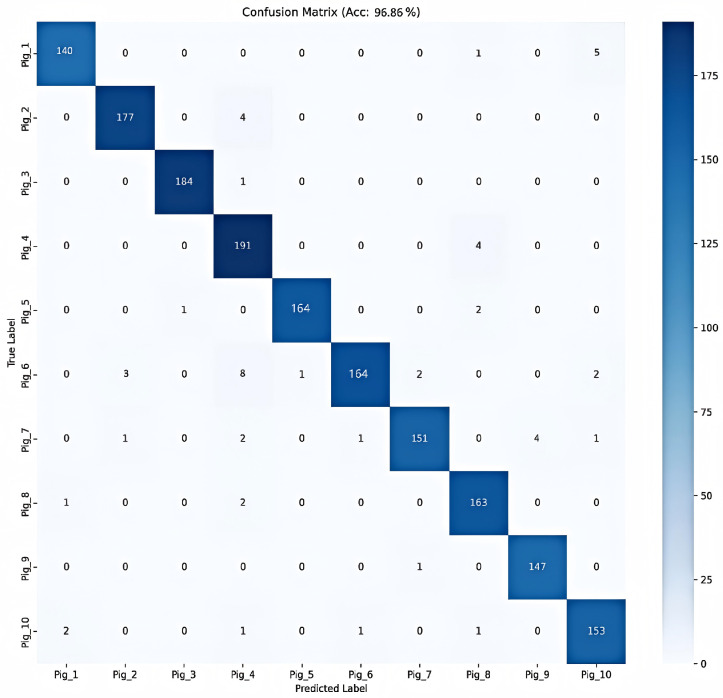
Confusion matrix analysis of the proposed DK-EffiPointMLP on the test dataset.

The granular performance of DK-EffiPointMLP is visualized via the confusion matrix in Figure 8. It is evident that the model performs exceptionally well across the dataset, with individuals like Pig_3 and Pig_4 showing nearly perfect recognition rates. While minor confusion is observed in specific cases—for instance, a few samples of Pig_6 being misclassified as Pig_4—the overall error distribution is sparse and randomized. This level of classification density on the diagonal effectively demonstrates that DK-EffiPointMLP provides a robust feature representation that minimizes inter-class interference, thereby supporting the reported OA of 96.86%.

To further assess the generalization capability of the model, we validated DK-EffiPointMLP on the public ModelNet40 dataset [35] and benchmarked it against representative methods, including PointNet, PointNet++, and PointMLP. The comparative results, presented in Table 4, demonstrate that the proposed feature extraction method exhibits competitive performance in general classification tasks.

ModelNet40 serves as a classic benchmark dataset for point cloud classification tasks, and testing on this public dataset was designed to evaluate the generalization and robustness of the proposed method. Experimental results demonstrate that the DK-EffiPointMLP model achieves superior performance, attaining an Overall Accuracy (OA) of 95.2% and a Mean Class Accuracy (mAcc) of 92.8% on the ModelNet40 dataset. Compared to the baseline PointMLP model (OA: 94.1%, mAcc: 91.5%), our approach achieves improvements of 1.1% and 1.3% in OA and mAcc, respectively. These results validate that the integration of the enhanced module significantly boosts the model’s classification performance across diverse point cloud data scenarios.

### 3.5. Ablation Experiment

To demonstrate that conventional 3D point cloud recognition methods generally suffer from low computational efficiency and limited feature representation capabilities, often failing to adequately capture local neighborhood structures while incurring substantial computational and memory overhead, comparisons were conducted using PointMLP as the baseline model. *Note:* ✓: Module included; ×: Module omitted. Detailed results are presented in Table 5.

### 3.6. Key Parameter Sensitivity Analysis (k Value)

Impact of Local Map k-Value Sizes. Similar to how Convolutional Neural Networks (CNNs) adjust the receptive field size by defining kernels of varying dimensions, in point cloud processing, the k-value determines the size of the extracted local receptive field, which in turn influences the generated local features. Consequently, comparative experiments were conducted with different k-values, while the number of groups was fixed at 8. As shown in Table 6, the classification accuracy is optimal when the k-value is set to 24 for each layer.

### 3.7. Analysis of Space and Time Complexity

Time and Space Complexity Analysis. Table 7 summarizes the spatial complexity (model parameter count) and temporal complexity (floating-point operations per sample, FLOPs). Here, ‘M’ denotes millions and ‘G’ denotes billions. The results indicate that the proposed DK-EffiPointMLP exhibits superior computational efficiency. It possesses significantly fewer parameters than the PointMLP baseline, demonstrating better storage efficiency while maintaining equivalent feature representation capabilities.

## 4. Discussion

This study addresses the critical need for non-contact individual identification in precision pig farming by proposing DK-EffiPointMLP, a method based on dual-stream local aggregation and PointMLP. Experimental results demonstrate that the proposed method achieves an overall accuracy of 96.86% on our self-constructed dataset, significantly outperforming existing baseline models. This outcome validates our core hypothesis that “integrating multi-scale neighborhood aggregation with channel-efficient attention can significantly enhance the model’s ability to capture subtle surface features.” The DLF module effectively overcomes the limitations of traditional single-scale approaches in handling complex pose variations by fusing multi-scale geometric information through a dual-branch strategy. Meanwhile, the EffiConv module strengthens key feature representations while reducing computational redundancy through the synergistic combination of PConv and the SE mechanism. From a broader perspective, the success of this study provides strong support for the application of MLP-based point cloud networks in agricultural vision tasks. Compared to the work of Zhou et al. [23], our multi-scale design demonstrates clear advantages in addressing uneven point cloud density and local feature loss. Additionally, the findings align with Saleem et al.’s perspective [29] on balancing performance and computational efficiency in network architecture optimization.

Compared to traditional tagging methods, this non-contact approach offers potential advantages in reducing labor intensity and minimizing animal stress, providing a new technological pathway for refined livestock management. However, this study still has several limitations that require attention. The reliance on specific scene settings for data collection restricts the generalizability of the method, and the manual intervention in the current preprocessing pipeline affects the system’s level of automation. Furthermore, the scale and diversity of the dataset need to be further expanded to comprehensively evaluate the model’s generalization ability across different breeds and farming environments.

Future research will focus on addressing the current limitations, including the labor-intensive manual preprocessing, the need to expand the sample size to cover a more diverse range of individuals and breeds, the requirement for multi-farm validation beyond single-device constraints, and further mitigation of temporal leakage. Building on this, exploring multi-modal information fusion strategies and edge computing optimization schemes can further enhance the robustness and practicality of the system in real farming environments. These steps aim to improve the model’s robustness for real-world smart farming and broader point cloud tasks, providing more reliable technical support for the deeper application of 3D point cloud-based biometric recognition technology in agricultural intelligence. As the technology continues to improve, such methods are expected to play an increasingly important role in promoting the digital transformation of the livestock industry.

## 5. Conclusions

This paper addresses the critical need for non-contact individual identification in precision pig farming by proposing DK-EffiPointMLP, a method for recognizing geometric features in 3D point clouds of pig backs based on dual-stream local aggregation and PointMLP. By incorporating a dual-branch local feature enhancement module, the model effectively balances local geometric details with a broad receptive field, thereby strengthening its ability to characterize curvature features of the dorsal region. Combining efficient Partial Convolutions (PConv) with SE attention mechanisms, it substantially suppresses channel redundancy while amplifying the representational strength of critical geometric features. Experiments demonstrate that the proposed method achieves an Overall Accuracy (OA) of 96.86% and a Mean Class Accuracy (mAcc) of 96.87% on our self-constructed dataset, significantly outperforming both PointNet++ and the original PointMLP baseline. This effectively unifies high precision with parameter efficiency.

## Figures and Tables

**Table 1:** Detailed architecture configuration of the proposed DK-EffiPointMLP.

Stage	Input Points (N)	Output Channels (C)	Blocks	K Neighbors	SE Ratio (r)
Stage 1	1024	64	2	24	8
Stage 2	512	128	2	24	8
Stage 3	256	256	2	24	8
Stage 4	128	512	2	24	8
Classifier	Global Avg Pooling + FC (512, 256) + FC (256, 10)				

**Table 2:** Experimental environment settings and training parameters.

Category	Item	Setting
Experimental Environment	Operating system	Ubuntu 20.04
	CPU	Intel Xeon Platinum 8270 @ 2.70 GHz
	Memory	64 GB
	GPU	NVIDIA GeForce RTX 4090 D (24 GB memory)
Training Parameters	Deep learning framework	PyTorch 1.1.0
	Programming language	Python 3.8
	Optimizer	SGD
	Nesterov	False
	Initial learning rate	0.0015
	Learning rate scheduler	Cosine Annealing
	Batch size	16
	Maximum epochs	150
	Weight decay	$5\times 10^{-4}$

**Table 3:** Comparison results of different methods on the pig individual identification dataset.

Method	OA (%)	mAcc (%)
PointNet	88.92	89.04
PointNet++	92.93	92.97
PointMLP	94.12	94.27
DK-EffiPointMLP (Our)	96.86	96.87

**Table 4:** Comparison results on the ModelNet40 dataset.

Method	OA (%)	mAcc (%)
PointNet	89.2	86.2
PointNet++	91.9	88.1
PointMLP	94.1	91.5
DK-EffiPointMLP (Our)	95.2	92.8

**Table 5:** Impact of module combinations on pig identification performance.

Model	Dual-Branch Feature Enhancement Module	EffiConv (PConv + SE)	OA (%)	mAcc (%)
A	×	×	94.12	94.27
B	✓	×	95.67	95.64
C	✓	✓	96.86	96.87

**Table 6:** Experimental results with different k values.

k-Value	OA (%)	mAcc (%)
16	96.33	96.35
20	96.55	96.58
24	96.86	96.87
28	96.43	96.45

**Table 7:** Space and time complexity analysis of different models.

Method	#Params	FLOPs/Sample
PointNet	3.5 M	0.44 G
PointNet++	1.48 M	0.86 G
PointMLP	13.41 M	31.4 G
DK-EffiPointMLP (Our)	13.10 M	60.2 G

## Data Availability

The dataset was developed by our research team and will be made publicly accessible upon reasonable request.
